# The role of radiomics with machine learning in the prediction of muscle-invasive bladder cancer: A mini review

**DOI:** 10.3389/fonc.2022.990176

**Published:** 2022-08-17

**Authors:** Xiaodan Huang, Xiangyu Wang, Xinxin Lan, Jinhuan Deng, Yi Lei, Fan Lin

**Affiliations:** Department of Radiology, The First Affiliated Hospital of Shenzhen University, Health Science Center, Shenzhen Second People’s Hospital, Shenzhen, China

**Keywords:** bladder cancer, radiomics, machine learning, muscle-invasive, CT, MRI

## Abstract

Bladder cancer is a common malignant tumor in the urinary system. Depending on whether bladder cancer invades muscle tissue, it is classified into non-muscle-invasive bladder cancer (NMIBC) and muscle-invasive bladder cancer (MIBC). It is crucial to accurately diagnose the muscle invasion of bladder cancer for its clinical management. Although imaging modalities such as CT and multiparametric MRI play an important role in this regard, radiomics has shown great potential with the development and innovation of precision medicine. It features outstanding advantages such as non-invasive and high efficiency, and takes on important significance in tumor assessment and laor liberation. In this article, we provide an overview of radiomics in the prediction of muscle-invasive bladder cancer and reflect on its future trends and challenges.

## 1 Introduction

Bladder cancer (BC) is the second most common cancer among urological malignancies, with an estimated 573,200 people diagnosed with BC worldwide in 2020 (1). The rates of bladder cancer increase with age. The risk of BC is multifactorial, with smoking (2) being the most important risk factor. Uroepithelial carcinoma accounts for approximately 90% of bladder cancer cases and typically presents as multifocal and recurrent; other subtypes are squamous cell carcinoma (6-8%) and adenocarcinoma (3).

Determining the invasion of the tumor into the muscle layer of the bladder wall is probably the most critical step in clinical management, as it directly affects the patient’s treatment strategy. Bladder cancers are classified into non-muscle-invasive bladder cancer (NMIBC) (≤ T1 stage) and muscle-invasive bladder cancer (MIBC) (≥ T2 stage) according to whether they invade muscle tissue or not. NMIBC is mostly in the early stages of the disease, with a 5-year probability of recurrence and progression of 78% and 45%, respectively (4), while MIBC has a poor prognosis, with approximately 50% (5) of patients developing metastases within 2 years after radical cystectomy(RC). NMIBC is usually treated by transurethral resection of bladder tumors(TURBT) with or without intravesical chemotherapy (6). Whereas MIBC is usually treated with radical cystectomy(RC), radiotherapy, chemotherapy, or combination therapy (5). Currently, pathological examination of TURBT specimens is the gold standard for identification of MIBC. However, according to previous studies, the error rate is about 20-80% due to problems such as differences in resection (7). Even though the error rate can be reduced by repeating TURBT, underestimation of staging and delayed treatment of the condition may lead to disease progression and worse prognosis, and this invasive operation also carries some safetyoperational risks. Faced with the above problems, scholars have searched for an alternative, non-invasive and efficient diagnostic tool to accurately predict muscle-invasive bladder cancer, so they have turned their attention to “radiomics” - a hot and promising diagnostic technology. Radiomics is the extraction and analysis of quantitative imaging features from imaging tools (CT, MRI, PET-CT, etc.) for the development of descriptive and predictive models (8). Machine learning (ML), a branch of artificial intelligence, is a typical approach used in radiomics model generation (9). Through the inferential training of datasets, ML aids in the development of highly accurate and effective predictive models based on radiomics analysis (10). In this paper, we review the current existing research related to our topic, summarize the results of using machine learning to accurately predict muscle-invasive bladder cancer, and reflect on the future directions and challenges of the topic.

## 2 Search criteria

A comprehensive review of current literature was performed using the PubMed-Medline and Web of Science database up to April 5, 2022 using “bladder cancer”, combined with one of the following terms: “radiomics”, “machine learning”, and “artificial intelligence” in combination with “muscle invasive”.

**Figure 1:**
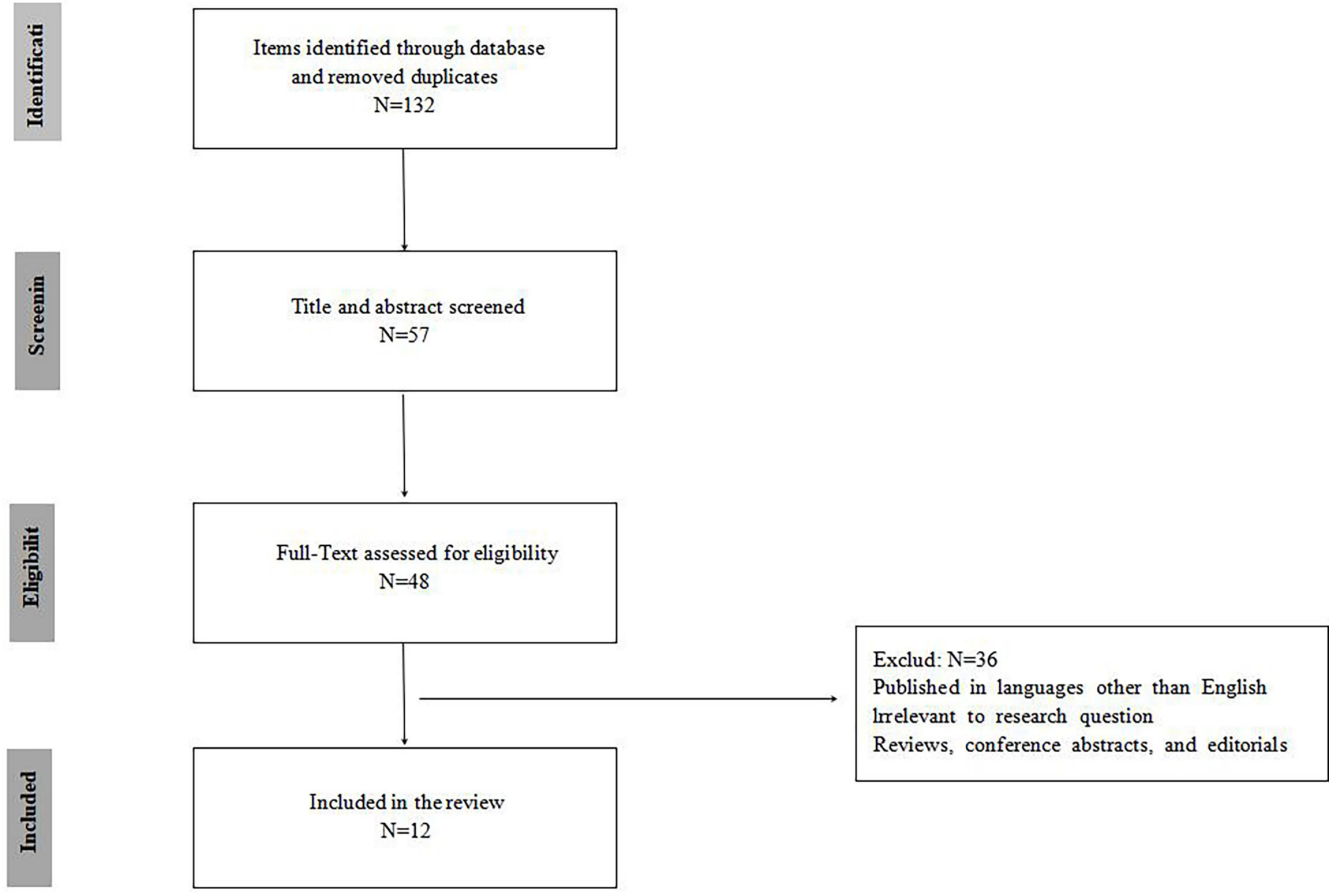
PRISMA flowchart of included studies.

The exclusion criteria for the articles were as follows:
(1) Published in a language other than English.
(2) The purpose of the article study was not to predict muscle invasion of bladder cancer.
(3) The article was not studied with imaging tools.
(4) Reviews, conference abstracts, and editorials were excluded.
The inclusion criteria for the article were as follows:
(1) Background introduction of radiomics, machine learning, deep learning or artificial intelligence and bladder cancer.
(2) The purpose of the article study was to predict muscle invasion of bladder cancer.
(3) The article was studied with imaging tools(CT, MRI, PET-CT, SPECT e.g.).
In accordance with the PRISMA criteria, **Figure 1** was included to delineate our article
selection process.

## 3 Results

The final collection of 12 relevant publications found that the first study started in 2017, reflecting the fact that radiomics is a relatively new concept in the field of BC. The literature related to machine learning for predicting muscle-invasive bladder cancer is summarized in **Table 1** (11–22). For studies in this field, four were based on enhanced CT and the remaining eight were related to MRI. Only 16.7% (2/12) of the studies were multi-center studies.

**Table 1:** Studies included in the systematic review.

	Study characteristics			Patient characteristics				Imaging characteristics	
	Author	Year	Study design	Number of cases	Number of selected lesions	Surgical technique	Pathological stage NMIBC: MIBC	Imaging modality	Scanner
1	Xu	2017	Single-center retrospective	78	118	NA	34:84	T2WI	3.0T GE
2	Garapati	2017	Single-center retrospective	76	84	Cystectomy	43:41	CTU	NA
3	Tong	2018	Single-center retrospective	65	65	Cystectomy	31:34	T2WI	1.5-3.0T
4	Xu	2019	Single-center retrospective	54	54	NA	24:30	T2WI、DWI、ADC	3.0T GE
5	Zheng	2019	Single-center retrospective	199	199	RC or TURBT	130:69	T2WI	3.0T MR scanner (Intera Achieva, Philips Medical Systems)
6	Xu	2020	Single-center retrospective	218	218	Both TURBT and RC	131:87	DWI	3.0T MR scanner (Ingenia;Philips Healthcare)
7	Wang	2020	Mult-center retrospective	106	106	RC or partial cystectomy or TURBT	64:42	T2WI、DWI、ADC	3.0T MR system (MAGNETOM Trio, Siemens Healthineers)
8	Hammouda	2021	Single-center retrospective	42		NA		T2WI、DWI、ADC	3.0T Ingenia Philips MRI scanners
9	Zhang	2021	Mult-center retrospective	441	441	RC or TURBT	183(development):110(tuning ) :73(internal validation) :75(external validation)	Enhanced CT	NA
10	Zheng	2021	Single-center retrospective	185	185	NA	129:56	T2WI、DCE	3.0T MRI scanner(Magnetom Verio: Siemens, Erlangen, Germany)
11	Zhou	2021	Single-center retrospective	100	100	NA	70:30	Enhanced CT	Siemens 64-row spiral CT
12	Cui	2022	Single-center retrospective	327	188	RC or partial cystectomy or TURBT	120:68	CECT	GE Dis covery CT750HD, GE LightSpeed VCT, Philips ICT 256, and Siemens Somatom Definition Flash.

ADC, apparent diffusion coeffificient; CECT, contrast-enhanced computed tomography; CT, computed tomography; CTU, CT Urography; DCE, dynamic contrast enhanced; DWI, diffusion-weighted imaging; MIBC, muscle-invasive bladder cancer; MR, magnetic resonance; MRI, magnetic resonance imaging; NA, not available; NMIBC, non–muscle-invasive bladder cancer; RC, radical cystectomy; TURBT, transurethral resection of bladder tumor; T2WI, T2-weighted imaging.

## 4 Discussion

### 4.1 Traditional diagnostic imaging

In current clinical practice, medical imaging techniques including CT, MRI and other non-invasive and safe diagnostic modalities are increasingly recognized for their performance in predicting muscle invasion and staging of bladder cancer. MRI has mainly been found to play a crucial role in the early localization and invasive diagnosis of BC. T2-weighted imaging(T2WI) is able to illustrate detailed structural information of the lesion and bladder wall, thus potentially reflecting the depth of invasion of the bladder wall of BC. The low signal line of the detrusor muscle is interrupted by MIBC, whereas the detrusor muscle is complete in NMIBC. Diffusion Weighted Imaging(DWI) and Apparent Diffusion Coefficient(ADC) have a good ability to reflect signal intensity differences between muscle, peritumoral inflammation and fibrosis (23, 24). The significance of dynamic contrast enhanced MRI(DCE-MRI) in assessing tumor aggressiveness depends on the neoangiogenesis of the tumor, which is an important factor in tumor growth; the more neovascularization there is, the higher the tumor stage and grade (25). In studies on dynamic enhancement sequences, the tumor, bladder mucosa and submucosa show early enhancement, but the bladder wall muscle maintains its low signal and delays enhancement. As early as 2000, Hayashi et al. observed that image signs of submucosal linear enhancement (SLE) at the base of the tumor were frequently seen on DCE images of NMIBC patients (26). This discovery is unquestionably a watershed moment in imaging-based BC staging and muscle-invasive status (MIS) diagnosis. Takeuchi et al (27) followed up by reporting an important feature found in most NMIBC on DWI, the tumor stalk, which improved the accuracy and robustness of imaging-based BC staging and MIS diagnosis. The accuracy of staging based on tumor stalk was 91.3% in Wang et al. study, while the accuracy of SLE staging was 91.3% (23). Panebianco et al (28) proposed Vesical Imaging-Reporting and Data System (VI-RADS) to quantify these signs on Multi-Parametric Magnetic Resonance Imaging (mpMRI) and to standardize the diagnostic procedure for image-based MIS prediction based on these features. This scoring system has effective diagnostic performance. In the Ueno et al. study, for example, the combined area under the curve(AUC) of five radiologists diagnosing MIBC was as high as 0.90 (29). Another prospective study also demonstrated the high diagnostic reliability of the VI-RADS score (AUC value of 0.94), especially for scores 1-2 and 3-5 (sensitivity 91.9%, 95%; specificity 91.1%, 95%) (30). The VI-RADS scoring method relies on expert visual perception judgment, yet it is still semi-qualitative. As a result, research into the objective and accurate radiomic detection of bladder cancer muscle invasion is required.

### 4.2 Radiomics

Radiomics is a relatively young concept, and Prof. Lambin originally described it in 2012 (31). Radiomics refers to the high-throughput extraction of image features from the region of interest (ROI) of radiological imaging techniques (CT, MR, but also PET, etc.) for automated analysis, using machine and deep learning techniques to extract critical information for accurate quantitative assessment of lesions, and ultimately for aiding in the diagnosis, classification, or grading of diseases. Radiomics inherits the technological benefits of reproducible, non-invasive radiological imaging over biopsy, making patient status monitoring and prognosis safer and more reliable.

Radiomics techniques can be classified into two groups: those using manual radiomics features and those using deep learning radiomics (32, 33) Traditional manual radiomics has the following four main processing tasks: image acquisition and preprocessing; image segmentation; feature extraction and quantification; and model building. The difference is that segmentation is not a necessity in the automated radiomics pipeline (33).. Radiomics has been increasingly studied in medical field for lung cancer, breast cancer, glioma, prostate cancer and other disorders (34–37). One of the current topics in bladder cancer research is the radiomics prediction of MIBC.

The pertinent radiomics literature is described below in terms of modality selection, volumes of interest (VOIs) segmentation, feature selection, model construction, and integration of clinical features, respectively.

#### 4.2.1 Input modality

It mainly based on enhanced CT, MRI, with MRI accounting for (8/12) of the included literature. Since CT is weaker than MRI in discriminating soft tissues and the borders and bases of lesions are rarely distinguishable in discriminating MIS (38), there is a greater preference for MRI, mainly around T2WI, DWI and ADC and DCE sequences. In 2017, Garapati (11) and Xu et al. (12)established a precedent for using radiomics to predict MIS using CT and MRI, respectively, and inspired readers to combine additional MRI sequences to improve the possibility of differentiation task performance. As a result, extensive research on the precise differentiation of NMIBC and MIBC using radiomic methods with multi-parametric MRI images started to be conducted. Xu et al. obtained mean accuracies of 79.63%, 81.37%, and 91.22% for T2WI, DWI, and the combined of both sequences, with AUCs of 0.8828, 0.8884, and 0.9756, respectively (14). The superiority of DWI sequences over T2WI sequences in reflecting heterogeneous differences between NMIBC and MIBC (14, 16) has been repeatedly demonstrated. This might be because muscle-infiltrating tumors have a propensity to impede water molecule diffusion by shrinking extracellular space (39–41), which is better captured by DWI and the related ADC maps. And multi-sequence MRI was more helpful to predict the muscle invasion condition of BC preoperatively compared with single sequence T2WI and DWI, which was consistent with previous knowledge.

#### 4.2.2 Volumes of interest segmentation

The three basic methods of delineating the area of interest are manual, semi-automated, and automatic. Even with computerized techniques, radiologists still need to examine and manually adjust them to assure the correctness of ROI descriptions because the majority of them are still primarily manual, which takes time and is tiresome. Initially, academics mostly concentrated on the overall tumor volume. As research developed, it was generally acknowledged that the information in the region around the tumor also held a lot of relevant information. The body of literature suggests that the determination of muscle invasiveness is related to bladder tumors as well as the tumor’s base (15) and adjacent bladder tissue (13). In addition, most of the relevant experiments have been conducted so far at the 3D level. Compared to 2D system analysis, 3D has higher precision and AUC (95.24% and 0.9864 *vs*. 92.86% and 0.9705) (18) which reflects the importance of 3D processing as it provides a comprehensive BC assessment with full descriptive information and details.

#### 4.2.3 Feature extraction and quantization

Currently, there are mainly shape and intensity features based on histogram, texture features including gray level co-occurrence matrix (GLCM), gray Level run length matrix (GLRLM), gray-level size zone matrix (GLSZM), gray level dependence matrix (GLDM), neighborhood gray-tone difference matrix (NGTDM), and higher-order feature wavelet features. The global, local and regional distribution features of image grayscale can be comprehensively described. Although there are a large number of features available for analysis, redundancy of features can seriously affect prediction performance. So feature selection is essential for developing optimal prediction models. Combined with other advanced selection strategies for statistical analysis, such as support vector machine (SVM)-based recursive feature elimination (SVM-RFE), the least absolute shrinkage and selection operator

(LASSO), max-relevance and min-redundancy(mRMR), these methods are widely used to reduce the impact of feature redundancy, and other methods such as Boruta are also used. After feature selection, Xu et al. found that the run length matrix (RLM) features accounted for a greater proportion of 13/19 in the optimal subset (14), better reflecting the regional heterogeneity differences between NMIBC and MIBC. The Co-occurrence matrices(CM), RLM and GLSZM features were found to be favorable feature classes for predicting BCa muscle invasion condition by Wang et al. (16).

#### 4.2.4 Model construction

Different machine learning classifiers can be employed with the chosen features to create predictive models. Classifiers that are typically used include LASSO, SVM, random forest (RF), logistic regression, etc. Convolutional neural networks (CNN) are the most commonly used artificial neural networks for deep learning. SVM-RFE was the most commonly used machine learning method (7/12), among all the methods used for the classification task. **Table 2** demonstrates how different models’ prediction efficacy varies. NN, SVM, and RF classifier diagnostic performance were tested by Hammouda et al. in descending order (18). Garapati et al. observed that the AUC for morphological and texture features was roughly 0.90 (11); for various other mri-based radiomics models, the AUC ranged from 0.87 to 0.98 (14–17). However, all of the preceding experiments have the disadvantage of lacking independent external validation, so the true validity of the diagnostic performance of these models must be confirmed further. In contrast, so far, the prediction model developed by Zhang et al. is the only experiment with external validation results. But the AUC (0.791-0.936) of the study by Zhang et al. was slightly lower (19). This may be the risk of misclassification of some models influenced by tumor size, which may lead to a decrease in the diagnostic performance of the model, and therefore tumor size is one of the critical features to determine the muscle invasion condition of BC.

**Table 2:** Radiomic characteristics of studies included in the systematic review.

Author	Segmentation method	Radiomic feature categories	Machine-learning method for feature selection	Number of selected features	Model	AUC of radiomic model with the best performance		clinical factor	AUC of radiomic-clinical model	
						**Training set**	**Validation set**		**Training set**	**Validation set**
Xu	Semi-automatic segmentation(3D)	Signal intensity histogram-based features and 3D ND-Haralick texture features based intensity and its high-order derivative maps	SVM-RFE","SMOTE	13	SVM-RFE	0.861	NA	NA	NA	NA
Garapati	Automatic segmentation(3D)	First-order statistics, shape, contrast, GLRLM,	Stepwise feature selection	3 subsets of radiomic features	LDA, NN, SVM, RF	0.97	NA	NA	NA	NA
Tong	Manual segmentation(3D)	LBP、GLCM	An optimal biomarker approach	9	SVM	Patient level:0.806,radial sector level:0.813	NA	NA	NA	NA
Xu	Manual segmentation(2D)	Histogram, CM , RLM,	SVM-RFE","SMOTE,	19	SVM-RFE	0.9857	NA	NA	NA	NA
Zheng	Semi-automatic segmentation(3D)	first-order statistics,shape-based,GLCM,GLRLM,GLSZM,NGTDM,and GLDM	LASSO LR	23	LASSO	0.913;Optimism-corrected:0.912	0.874	Tumor size	0.922;optimism-corrected AUC of 0.921	0.876
Xu	Manual segmentation and automatic segmentation(3D)	First-order intensity features,high-order texture features,and shape ,GLCM,GLRLM,GLSZM and NGTDM	Boruta	21	RF, AR	.0.907	0.904	RandomForest model and TURBT	NA	NA
Wang	Manual segmentation and automatic segmentation(2D)	Histogram , CM, RLM, NGTDM and GLSZM	SVM-RFE	36	LR, LASSO	0.88	external validation cohort 0.813	Radscore and tumor stalk	0.924	0.877
Hammouda	Automatic segmentation(3D)	Histogram ,GLCM,GLRLM,and morphological features	NA	NA	NN(best)","RF,SVM	0.9864		NA	NA	NA
Zhang	Semi-automatic segmentation(3D)	NA	NA	NA	FGP-Net	development cohort:0.936, tuning cohort:0.891	internal validation cohort: 0.861,external validation cohort: 0.791	NA	NA	NA
Zheng	manual segmentation (3D)	shape and size-based features, image intensity, textural features and wavelet features	mRMR	40	Lasso(best)、SVM、RF	0.934	0.906	VI-RADS	0.97	0.943
Zhou	semi-automatic segmentation(3D)	GLDM,Shape2D,GLCM,Shape3D,First-order,GLRLM,GLSZM,and NGTDM	SVM-RFE	6	LR, Decision Tree, SVM(best), and Adaboost algorithm	0.898	0.702	Rad-score, albuminuria and metabolic syndrome	0.8457	

AR, all-relevant model; AUC, area under the curve; CM, Co-occurrence matrices; 3D, three dimensional; 2D, two dimensional; FGP-Net, Filter-guided Pyramid Network; GLCM, grey-level co-occurrence matrix; GLDM, gray level dependence matrix; GLRLM, gray-level run length matrix; GLSZM, gray-level size zone matrix; LASSO, least absolute shrinkage and selection operator; LBP, local binary pattern; LDA, linear discriminant analysis; LR, logistic regression; mRMR, min-redundancy; NA, not available; ND, nondirectional Haralic textural features; NN, neural network; NGTDM, neighborhood gray-tone difference matrix; RF, random forest model; RFE, recursive feature elimination; RLM, run length matrix; SMOTE, synthetic minority oversampling technique; SVM, support vector machine classififier; TURBT, transurethral resection of bladder tumor.

#### 4.2.5 Integration of other clinical factors

It has become a trend to include clinical risk factors in the prediction model in order to better predict MIS and improve clinical diagnostic performance and application value. These include tumor size (15), tumor stalk (16), proteinuria and multiple sclerosis (21), as well as VI-RADS (20) and TURBT (14).The radiomic model incorporating clinical factors performed significantly better than the conventional MRI examination and simply radiomic model in terms of calibration and discrimination. Radiomic-clinical nomogram can be used as a reliable and non-invasive adjunct to differentiate MIBC from NMIBC preoperatively (15).

#### 4.2.6 Method for validating results

83.3 percent (10/12) of the retrieved literature were single-center studies (11–15, 17, 18, 20–22), and the internal validation method was primarily used for model validation. Only two paper performing external validation of the results (16, 19). Because of the lack of externally validated results, the reliability of the remaining articles’ results in terms of diagnostic efficacy is questionable. The sensitivity, specificity, and AUC of the internal validation cohort in Zhang’s prediction model were 0.733, 0.810, and 0.861, respectively, while those of the external validation cohort were 0.710, 0.773, and 0.791, respectively (19).

## 5 Future and prospects

Of these 12 studies, all were retrospective, subject to selection bias and prone to data loss. Because the sample size was insufficient, cross-validation was essentially required to make up for it. Additionally, only two of the results were externally validated using radiomics models, with the rest being single-center, internally validated results that were not convincing. The current radiomics models are mainly based on single-modality or dual-modality MRI, and there is no multi-modality study combining the three sequences of “T2WI, DWI and DCE”, which needs to be further validated to improve the differentiation performance. Therefore, investigations should be planned in a more thorough and subtle manner for a variety of therapeutic applications to increase the reliability of the results. To completely understand the diagnostic usefulness of machine learning in predicting MIBC, more prospective multi-center and various machine trials will be required in the future. In addition, for future optimization of this new approach, more studies are needed to test the potential of optimizing predictive models by combining imaging biomarkers with other non-imaging biomarkers, such as urine and serum biomarkers. Although there have been significant advances in a number of studies, from fundamental tumor identification to precise staging and grading, recent research has also been gradually moving toward the prediction of treatment outcomes. The needs of the clinical market can no longer be met by illness diagnosis alone. After a bladder cancer diagnosis, increasing focus will be placed on how well machine learning predicts the response to treatment and prognosis outcome of the disease In the future.

## Author contributions

FL and YL contributed to the conception of the study. XW contributed significantly to analysis and manuscript preparation. XH performed the data analyses and wrote the manuscript. XL and JD organized and drew tables. All authors contributed to the article and approved the submitted version.

## Funding

This study is supported by China International Medical Foundation (No. Z-2014-07-2101).

## Conflict of interest

The authors declare that the research was conducted in the absence of any commercial or financial relationships that could be construed as a potential conflict of interest.

## Publisher’s note

All claims expressed in this article are solely those of the authors and do not necessarily represent those of their affiliated organizations, or those of the publisher, the editors and the reviewers. Any product that may be evaluated in this article, or claim that may be made by its manufacturer, is not guaranteed or endorsed by the publisher.

## References

[1] Sung H Ferlay J Siegel RL Laversanne M Soerjomataram I Jemal A. Global cancer statistics 2020: GLOBOCAN estimates of incidence and mortality worldwide for 36 cancers in 185 countries. CA Cancer J Clin (2021) 71(3):209–49. doi: 10.3322/caac.21660 33538338

[2] Chavan S Bray F Lortet-Tieulent J Goodman M Jemal A. International variations in bladder cancer incidence and mortality. Eur Urol (2014) 66(1):59–73. doi: 10.1016/j.eururo.2013.10.001 24451595

[3] Verma S Rajesh A Prasad SR Gaitonde K Lall CG Mouraviev V. Urinary bladder cancer: Role of MR imaging. Radiographics (2012) 32(2):371–87. doi: 10.1148/rg.322115125 22411938

[4] Sylvester RJ van der Meijden AP Oosterlinck W Witjes JA Bouffioux C Denis L. Predicting recurrence and progression in individual patients with stage Ta T1 bladder cancer using EORTC risk tables: a combined analysis of 2596 patients from seven EORTC trials. Eur Urol (2006) 49(3):466–5; discussion 475-7. doi: 10.1016/j.eururo.2005.12.031 16442208

[5] Sherif A Jonsson MN Wiklund NP. Treatment of muscle-invasive bladder cancer. Expert Rev Anticancer Ther (2007) 7(9):1279–83. doi: 10.1586/14737140.7.9.1279 17892428

[6] Josephson D Pasin E Stein JP. Superficial bladder cancer: Part 2. management. Expert Rev Anticancer Ther (2007) 7(4):567–81. doi: 10.1586/14737140.7.4.567 17428176

[7] Turker P Bostrom PJ Wroclawski ML van Rhijn B Kortekangas H Kuk C. Upstaging of urothelial cancer at the time of radical cystectomy: Factors associated with upstaging and its effect on outcome. BJU Int (2012) 110(6):804–11. doi: 10.1111/j.1464-410X.2012.10939.x 22321341

[8] Ferro M de Cobelli O Musi G Del Giudice F Carrieri G Busetto GM. Radiomics in prostate cancer: An up-to-date review. Ther Adv Urol (2022) 14:17562872221109020. doi: 10.1177/17562872221109020 35814914 PMC9260602

[9] Tătaru OS Vartolomei MD Rassweiler JJ Virgil O Lucarelli G Porpiglia F. Artificial intelligence and machine learning in prostate cancer patient management-current trends and future perspectives. Diagnostics (Basel) (2021) 11(2):354. doi: 10.3390/diagnostics11020354 33672608 PMC7924061

[10] Ge L Chen Y Yan C Zhao P Zhang P A R. Study progress of radiomics with machine learning for precision medicine in bladder cancer management. Front Oncol (2019) 9:1296. doi: 10.3389/fonc.2019.01296 31850202 PMC6892826

[11] Garapati SS Hadjiiski L Cha KH Chan HP Caoili EM Cohan RH. Urinary bladder cancer staging in CT urography using machine learning. Med Phys (2017) 44(11):5814–23. doi: 10.1002/mp.12510 PMC5689080 28786480

[12] Xu X Liu Y Zhang X Tian Q Wu Y Zhang G. Preoperative prediction of muscular invasiveness of bladder cancer with radiomic features on conventional MRI and its high-order derivative maps. Abdom Radiol (NY) (2017) 42(7):1896–905. doi: 10.1007/s00261-017-1079-6 28217825

[13] Tong Y Udupa JK Wang C Chen J Venigalla S Guzzo TJ. Radiomics-guided therapy for bladder cancer: Using an optimal biomarker approach to determine extent of bladder cancer invasion from t2-weighted magnetic resonance images. Adv Radiat Oncol (2018) 3(3):331–8. doi: 10.1016/j.adro.2018.04.011 PMC6128093 30202802

[14] Xu X Zhang X Tian Q Wang H Cui LB Li S. Quantitative identification of nonmuscle-invasive and muscle-invasive bladder carcinomas: A multiparametric MRI radiomics analysis. J Magn Reson Imaging (2019) 49(5):1489–98. doi: 10.1002/jmri.26327 30252978

[15] Zheng J Kong J Wu S Li Y Cai J Yu H. Development of a noninvasive tool to preoperatively evaluate the muscular invasiveness of bladder cancer using a radiomics approach. Cancer (2019) 125(24):4388–98. doi: 10.1002/cncr.32490 31469418

[16] Wang H Xu X Zhang X Liu Y Ouyang L Du P. Elaboration of a multisequence MRI-based radiomics signature for the preoperative prediction of the muscle-invasive status of bladder cancer: a double-center study. Eur Radiol (2020) 30(9):4816–27. doi: 10.1007/s00330-020-06796-8 32318846

[17] Xu S Yao Q Liu G Jin D Chen H Xu J. Combining DWI radiomics features with transurethral resection promotes the differentiation between muscle-invasive bladder cancer and non-muscle-invasive bladder cancer. Eur Radiol (2020) 30(3):1804–12. doi: 10.1007/s00330-019-06484-2 31773297

[18] Hammouda K Khalifa F Soliman A Ghazal M El-Ghar MA Badawy MA. A multiparametric MRI-based CAD system for accurate diagnosis of bladder cancer staging. Comput Med Imaging Graph (2021) 90:101911. doi: 10.1016/j.compmedimag.2021.101911 33848756

[19] Zhang G Wu Z Xu L Zhang X Zhang D Mao L. Deep learning on enhanced CT images can predict the muscular invasiveness of bladder cancer. Front Oncol (2021) 11:654685. doi: 10.3389/fonc.2021.654685 34178641 PMC8226179

[20] Zheng Z Xu F Gu Z Yan Y Xu T Liu S. Combining multiparametric MRI radiomics signature with the vesical imaging-reporting and data system (VI-RADS) score to preoperatively differentiate muscle invasion of bladder cancer. Front Oncol (2021) 11:619893. doi: 10.3389/fonc.2021.619893 34055600 PMC8155615

[21] Zhou Q Zhang Z Ang X Zhang H Ouyang J. A nomogram combined with radiomics features, albuminuria, and metabolic syndrome to predict the risk of myometrial invasion of bladder cancer. Transl Cancer Res (2021) 10(7):3177–91. doi: 10.21037/tcr-21-426 PMC8797668 35116625

[22] Cui Y Sun Z Liu X Zhang X Wang X. CT-based radiomics for the preoperative prediction of the muscle-invasive status of bladder cancer and comparison to radiologists' assessment. Clin Radiol (2022) 77(6):e473–82. doi: 10.1016/j.crad.2022.02.019 35367051

[23] Wang HJ Pui MH Guo Y Yang D Pan BT Zhou XH. Diffusion-weighted MRI in bladder carcinoma: the differentiation between tumor recurrence and benign changes after resection. Abdom Imaging (2014) 39(1):135–41. doi: 10.1007/s00261-013-0038-0 24072383

[24] Wang HJ Pui MH Guan J Li SR Lin JH Pan B. Comparison of early submucosal enhancement and tumor stalk in staging bladder urothelial carcinoma. AJR Am J Roentgenol (2016) 207(4):797–803. doi: 10.2214/AJR.16.16283 27505309

[25] Abouelkheir RT Abdelhamid A Abou El-Ghar M El-Diasty T. Imaging of bladder cancer: Standard applications and future trends. Medicina (Kaunas) (2021) 57(3):220. doi: 10.3390/medicina57030220 33804350 PMC8000909

[26] Hayashi N Tochigi H Shiraishi T Takeda K Kawamura J. A new staging criterion for bladder carcinoma using gadolinium-enhanced magnetic resonance imaging with an endorectal surface coil: A comparison with ultrasonography. BJU Int (2000) 85(1):32–6. doi: 10.1046/j.1464-410x.2000.00358.x 10619941

[27] Takeuchi M Sasaki S Ito M Okada S Takahashi S Kawai T. Urinary bladder cancer: Diffusion-weighted MR imaging–accuracy for diagnosing T stage and estimating histologic grade. Radiology (2009) 251(1):112–21. doi: 10.1148/radiol.2511080873 19332849

[28] Panebianco V Narumi Y Altun E Bochner BH Efstathiou JA Hafeez S. Multiparametric magnetic resonance imaging for bladder cancer: Development of VI-RADS (Vesical imaging-reporting and data system). Eur Urol (2018) 74(3):294–306. doi: 10.1016/j.eururo.2018.04.029 29755006 PMC6690492

[29] Ueno Y Takeuchi M Tamada T Sofue K Takahashi S Kamishima Y. Diagnostic accuracy and interobserver agreement for the vesical imaging-reporting and data system for muscle-invasive bladder cancer: A multireader validation study. Eur Urol (2019) 76(1):54–6. doi: 10.1016/j.eururo.2019.03.012 30922688

[30] Del Giudice F Barchetti G De Berardinis E Pecoraro M Salvo V Simone G. Prospective assessment of vesical imaging reporting and data system (VI-RADS) and its clinical impact on the management of high-risk non-muscle-invasive bladder cancer patients candidate for repeated transurethral resection. Eur Urol (2020) 77(1):101–9. doi: 10.1016/j.eururo.2019.09.029 31699526

[31] Lambin P Rios-Velazquez E Leijenaar R Carvalho S van Stiphout RG Granton P. Radiomics: extracting more information from medical images using advanced feature analysis. Eur J Cancer (2012) 48(4):441–6. doi: 10.1016/j.ejca.2011.11.036 PMC4533986 22257792

[32] Bera K Braman N Gupta A Velcheti V Madabhushi A. Predicting cancer outcomes with radiomics and artificial intelligence in radiology. Nat Rev Clin Oncol (2022) 19(2):132–46. doi: 10.1038/s41571-021-00560-7 PMC9034765 34663898

[33] Goldenberg SL Nir G Salcudean SE. A new era: artificial intelligence and machine learning in prostate cancer. Nat Rev Urol (2019) 16(7):391–403. doi: 10.1038/s41585-019-0193-3 31092914

[34] Smith CP Czarniecki M Mehralivand S Stoyanova R Choyke PL Harmon S. Radiomics and radiogenomics of prostate cancer. Abdom Radiol (NY) (2019) 44(6):2021–9. doi: 10.1007/s00261-018-1660-7 29926137

[35] Tagliafico AS Piana M Schenone D Lai R Massone AM Houssami N. Overview of radiomics in breast cancer diagnosis and prognostication. Breast (2020) 49:74–80. doi: 10.1016/j.breast.2019.10.018 31739125 PMC7375670

[36] Choi YS Bae S Chang JH Kang SG Kim SH Kim J. Fully automated hybrid approach to predict the IDH mutation status of gliomas *via* deep learning and radiomics. Neuro Oncol (2021) 23(2):304–13. doi: 10.1093/neuonc/noaa177 PMC7906063 32706862

[37] Kolinger GD García DV Kramer GM Frings V Zwezerijnen GJC Smit EF. Effects of tracer uptake time in non-small cell lung cancer (18)F-FDG PET radiomics. J Nucl Med (2022) 63(6):919–24. doi: 10.2967/jnumed.121.262660 PMC9157719 34933890

[38] Xu X. Study progress of noninvasive imaging and radiomics for decoding the phenotypes and recurrence risk of bladder cancer. Front Oncol (2021) 11:704039. doi: 10.3389/fonc.2021.704039 34336691 PMC8321511

[39] Hafeez S Huddart R. Advances in bladder cancer imaging. BMC Med (2013) 11:104. doi: 10.1186/1741-7015-11-104 23574966 PMC3635890

[40] Mannelli L Nougaret S Vargas HA Do RK. Advances in diffusion-weighted imaging. Radiol Clin North Am (2015) 53(3):569–81. doi: 10.1016/j.rcl.2015.01.002 PMC4979318 25953290

[41] Zhang X Xu X Tian Q Li B Wu Y Yang Z. Radiomics assessment of bladder cancer grade using texture features from diffusion-weighted imaging. J Magn Reson Imaging (2017) 46(5):1281–8. doi: 10.1002/jmri.25669 PMC5557707 28199039

